# Hypoxia-regulated carbonic anhydrase IX (CAIX) protein is an independent prognostic indicator in triple negative breast cancer

**DOI:** 10.1186/s13058-022-01532-0

**Published:** 2022-06-03

**Authors:** Chong Hui Clara Ong, Dong Yeul Lee, Bernett Lee, Huihua Li, Jeffrey Chun Tatt Lim, Johnathan Xiande Lim, Joe Poh Sheng Yeong, Hiu Yeung Lau, Aye Aye Thike, Puay Hoon Tan, Jabed Iqbal

**Affiliations:** 1 Histopathology Laboratory, Department of Anatomical Pathology, Singapore General Hospital, 20 College Road, Academia, Level 10, Diagnostics Tower, Singapore, 169856 Singapore; 2 School of Biological Sciences, Nanyang Technological University, 60 Nanyang Dr, Singapore, 637551 Singapore; 3 Agency of Science, Technology and Research (A*STAR), Singapore Immunology Network (SIgN), 8A Biomedical Grove Level 3 & 4. Immunos Building138648, Singapore, Singapore; 4 Division of Medicine, Singapore General Hospital, Singapore General Hospital, Outram Road, Singapore, 169608 Singapore; 5 Centre for Quantitative Medicine, Duke-NUS Medical School, 8 College Rd, Singapore, 169857 Singapore; 6 Agency of Science, Technology and Research (A*STAR), Institute of Molecular Cell Biology (IMCB), 61 Biopolis Dr, Singapore, 138673 Singapore; 7 Duke-NUS Medical School, 8 College Rd, Singapore, 169857 Singapore

**Keywords:** Triple-negative breast cancer, Hypoxia, Carbonic anhydrase IX, TNBC, CAIX, Prognosis, Hypoxia gene panel

## Abstract

**Background:**

The effect of extracellular microenvironment (hypoxia and pH) has been regarded as a key hallmark in cancer progression. The study aims to investigate the effects of carbonic anhydrase IX (CAIX), a key hypoxia-inducible marker, in triple-negative breast cancer (TNBC) in correlation with clinicopathological parameters and predicting survival outcomes.

**Methods:**

A total of 323 TNBC cases diagnosed at the Department of Anatomical Pathology, Singapore General Hospital from 2003 to 2013 were used. Immunohistochemical staining (IHC) was performed using CAIX antibody and digital mRNA quantification was performed using NanoString assays. CAIX membranous expression was correlated with clinicopathological parameters using Chi-squared test or Fisher’s exact tests. Disease-free survival (DFS) and overall-survival (OS) were estimated using Kaplan–Meier analysis and compared between groups with the log-rank test.

**Results:**

Forty percent of TNBCs were observed to express CAIX protein and demonstrated significant association with larger tumour size (*P* = 0.002), higher histological grade (*P* < 0.001), and significantly worse disease-free survival (DFS) and overall survival (OS) (after adjustment: HR = 2.99, 95% CI = 1.78–5.02, *P* < 0.001 and HR = 2.56, 95% CI = 1.41–4.65, *P* = 0.002, respectively). Gene ontology enrichment analysis revealed six significantly enriched cellular functions (secretion, cellular component disassembly, regulation of protein complex assembly, glycolytic process, cellular macromolecular complex assembly, positive regulation of cellular component biogenesis) associated with genes differentially expressed (*CAIX*, *SETX, WAS, HK2, DDIT4, TUBA4α, ARL1*). Three genes (*WAS, SETX *and* DDIT4*) were related to DNA repair, indicating that DNA stability may be influenced by hypoxia in TNBC.

**Conclusions:**

Our results demonstrate that CAIX appears to be a significant hypoxia-inducible molecular marker and increased CAIX protein levels are independently associated with poor survival in TNBC. Identification of CAIX-linked seven gene-signature and its relationship with enriched cellular functions further support the implication and influence of hypoxia-mediated CAIX expression in TNBC tumour microenvironment.

**Supplementary Information:**

The online version contains supplementary material available at 10.1186/s13058-022-01532-0.

## Background

Triple-negative breast cancer (TNBC) is an aggressive subtype of breast cancer with high five year mortality which is partly due to the lack of therapeutic target specificity on common breast cancer receptors such as oestrogen receptor (ER), progesterone receptor (PR) or the human epidermal growth factor receptor 2 (HER2) [1]. Further classification of TNBCs can be grouped into four molecular subgroups, driving many studies focusing on immunotherapy and new development in endocrine targeted treatments to identify potential targeted therapies [2]. Hypoxic microenvironment in tumour cells occurs in most solid malignancies, evolving tumours into an aggressive oncogenic metabolism, increasing metastasis and enhancing resistance to clinical therapies [3–5]. Studies have also shown that hypoxia markers such as hypoxia inducible factor 1 (HIF-1) and hypoxia-driving factors are associated poorly in TNBC outcomes [6–8].

HIF-1 is a heterodimeric protein composed of a constitutively expressed HIF-1ß subunit and an O_2_- regulated HIF-1α subunit [9, 10]. Increased HIFα activates target genes involved in tumour proliferation, angiogenesis, metabolism, apoptosis and metastasis [4]. Additionally, HIFα and its regulated proteins including carbonic anhydrase nine (CAIX) and glucose transporter 1 (GLUT1) are highly expressed in several type of cancers and are associated with dismal prognosis [11–14]. HIF-1 regulates key aspects of cancer biology, including pH regulation in glycolysis, through CAIX [15]. Over-expression of CAIX was observed in several solid tumours, and its link with invasiveness has given rise to the hypothesis that CAIX expression may contribute to advanced disease and tumour progression [11, 15]. Increased CAIX expression has been shown to be more common in TNBC compared to other subtypes of breast cancer and a marker of poor prognosis [11, 16]. Therefore, we investigated the impact of hypoxia-dependent CAIX in both protein and transcriptional expression on TNBC biology and outcome in order to elucidate its potential role as a therapeutic target in a subset of TNBC patients.

## Methods

### Study design and clinicopathological parameters

A total of 323 archival formalin-fixed paraffin-embedded (FFPE) TNBC specimens from patients diagnosed between 2003 and 2013 at the Department of Anatomical Pathology, Singapore General Hospital were analysed. 17 cases were excluded due to depleted tumour regions and/or IHC staining artefacts. Only IHC-proven invasive TNBC immunophenotype in female patients was included in the study while those with history of neoadjuvant chemotherapy, radiotherapy, and concomitant cancers were excluded. Clinicopathological parameters were reviewed (Tables 1, 2). The Centralized Institutional Review Board of SingHealth provided ethical approval for the retrospective study.

**Table 1:** Comparison of clinicopathological features of TNBC patients bearing positive or negative CAIX tumour cell expression

Factors	CAIX		
	CAIX negative	CAIX positive	*P* value
Age at diagnosis^a^ (years)	55.5 (47, 63)	55 (44, 61.8)	0.230
Ethnicity			0.617
Chinese	153 (84.1%)	93 (78.8%)	
Indian	9 (4.9%)	8 (6.8%)	
Malay	9 (4.9%)	6 (5.1%)	
Others	11 (6%)	11 (9.3%)	
Laterality			0.642
Left	98 (52.4%)	60 (49.2%)	
Right	89 (47.6%)	62 (50.8%)	
Histological grade			< 0.001*
1/2	40 (21.7%)	8 (6.6%)	
3	144 (78.3%)	114 (93.4%)	
Tumour size 20 mm			0.002*
≤ 20 mm	70 (38.7%)	25 (21.4%)	
> 20 mm	111 (61.3%)	92 (78.6%)	
Lymphovascular invasion			0.058
No	122 (68.9%)	64 (57.7%)	
Yes	55 (31.1%)	47 (42.3%)	
Lymph node positivity			0.072
Absent	84 (62.7%)	44 (50.0%)	
Present	50 (37.3%)	44 (50.0%)	
Tumour borders			0.112
Infiltrative	131 (97.0%)	78 (91.8%)	
Pushing	4 (3.0%)	7 (8.2%)	

^*^Statistically significant values (*P* < 0.05)

^a^Age is presented as median (Interquartile range)

**Table 2:** Correlation between CAIX tumour cell expression and HIF-1α tumour cell expression in triple-negative breast cancer

CAIX	HIF-1α		*P* value
	HIF-1α negative	HIF-1α positive	
CAIX negative	99 (67.8%)	66 (52.4%)	0.013*
CAIX positive	47 (32.2%)	60 (47.6%)	

^*^Statistically significant values (*P* < 0.05)

### Tissue microarray (TMA) construction

Tissue Microarray (TMA) was constructed as previously described [17], using tumour regions which was selected based on pathological assessment of > 50% of the sample being tumour area.

### Immunohistochemistry and immunoscoring

Tissue microarray sections of 4 µm thickness were cut onto Bond Plus slides (Leica Biosystems Richmond) and heated at 60 °C for 20 min. The slides were then incubated with primary antibodies specific for HIF-1α (rabbit monoclonal, Abcam, Cambridge, MA, US, diluted 1:200) and CAIX (rabbit monoclonal, Cell Marque, Rocklin, CA, USA, diluted 1:100) using Leica Bond Max autostainer (Leica Biosystems Melbourne) and Roche Ventana Benchmark Ultra (Ventana Medical Systems Arizona), respectively. Details of antibodies, labelling patterns and dilution factors can be found in Additional file 1: Table S1. Positive controls used for HIF-1α include glioblastoma and tonsil tissue, while renal cell carcinoma tissue was used as a positive control for CAIX. Antibodies were detected with diaminobenzidine substrate (DAB) as the chromogen, and counterstained with hematoxylin.

Immunoscoring was done by two trained pathologists to determine the staining intensity and percentage of tumour cells stained in each TMA core. Semi-quantitative H-score was used and calculated using intensity and percentage expressed, respectively. The H-score was calculated as follows: (3 × % strong staining) + (2 × % moderate staining) + (1 × % weak staining). To analyse HIF-1α expression, only homogenously and darkly stained nuclei were considered, and a median H-score of ≥ 1 was considered positive. The staining of CAIX was scored as positive using a median H-score of ≥ 1 for membrane staining. Tumours were then categorized into “CAIX-negative” and “CAIX-positive” subsets based on the median H-score of ≥ 1.

### RNA extraction and NanoString gene expression measurement

RNA was extracted from four FFPE sections of 10 µm thickness using the RNeasy FFPE kit (Qiagen, Hilden, Germany) on a QIAcube automated sample preparation system (Qiagen, Hilden, Germany), and was quantified by an Agilent 2100 Bioanalyzer system (Agilent, Santa Clara, CA, USA). A total of 100 ng of functional RNA (> 300 nucleotides) was assayed on the nCounter MAX Analysis System (NanoString Technologies, Seattle, WA, USA). The NanoString counts were normalized using the positive control probes as well as the housekeeping genes, as previously reported [18]. The count data were then logarithmically transformed prior to further analysis. A total of 386 genes in the NanoString panel were tested for significant differences between CAIX positive and CAIX negative groups.

### Gene ontology (GO) enrichment analysis

Seven genes that were significantly differentially expressed were analysed for gene ontology (GO) enrichment using an R package (topGO) and stringent selection criteria to avoid false positive results to effectively cluster functional genes into different biological processes. Significant ontology terms were determined by a *P* value < 0.05 in this study.

### Follow-up and statistical analysis

Follow-up data were obtained from electronic medical records. Disease-free survival (DFS) and overall-survival (OS) were defined as the time from diagnosis to recurrence or death/date of last follow-up, respectively.

Statistical analysis was performed using SPSS for Windows, Version 15. The relationship between the association the clinicopathological parameters and hypoxia-related protein biomarkers was tested using Chi-square test or Fisher’s exact test. Survival outcomes were estimated with the Kaplan–Meier analysis and compared between subgroups with the log-rank statistics. Multivariate Cox Regression was carried out to evaluate the effect of CAIX tumour cell expression level with survival adjusted to the effects of age, grade, tumour size, lymph node stage, lymph node positivity and/or HIF-1α H score; multivariate analysis was also carried out on combinatorial CAIX/HIF1α tumour cell expression level with survival adjusted to the effects of age, grade, tumour size and lymph node stage.

Genes that were significantly differentially expressed between the two sample groups (positive-CAIX, negative-CAIX) were identified using Student t-tests with Welch’s correction and was used to determine differentially expressed genes (DEGs). Multiple testing corrections were applied using the method of Benjamini and Hochberg. The selection of seven significantly differentially expressed genes was based on statistical significance (*P* < 0.05) using t-tests (on the expression values) and multiple testing corrections (method of Benjamini and Hochberg), as seen in Additional file 1: Figure S1. Hierarchical clustering using complete linkage on Euclidean distances for both samples and genes generated a heat map, and is coloured by the gene expression levels (log2 counts) which has been mean centred and scaled by standard deviation on a per gene basis with the highest expression in red and the lowest expression in blue (Fig. 4).

All gene expression and survival data for the Molecular Taxonomy of Breast Cancer International Consortium (METABRIC) and The Cancer Genome Atlas (TCGA) were obtained from cBioPortal (http://www.cbioportal.org/) [19–21]. Statistical significance was defined by *P* value < 0.05.

## Results

### Positive CAIX membrane staining is associated with larger tumour size, higher histological grade and poorer survival rates

**Fig. 1:**
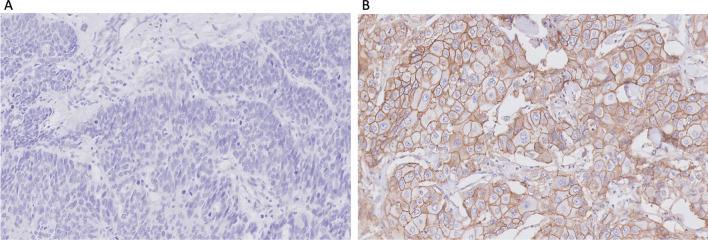
Representative immunohistochemical staining of **A** negative and **B** positive CAIX tumour cell expression in TNBC sections

Positive CAIX membranous staining in tumour cells was present in approximately 39.5% of the TNBC cohort (121/306) (Fig. 1). Approximately 45.9% of the tumour showed HIF-1α expression (141/307). However, the expression was variable throughout the tumour with some accentuation near areas of necrosis.

Significant associations were found between CAIX positivity in tumour cells and clinicopathological features such as larger tumour size (*P* = 0.002) and higher histological grade (*P* < 0.001) in Table 1. However, positive HIF-1α expression did not show any significant association with any clinicopathological parameters (Additional file 1: Table S2).

**Fig. 2:**
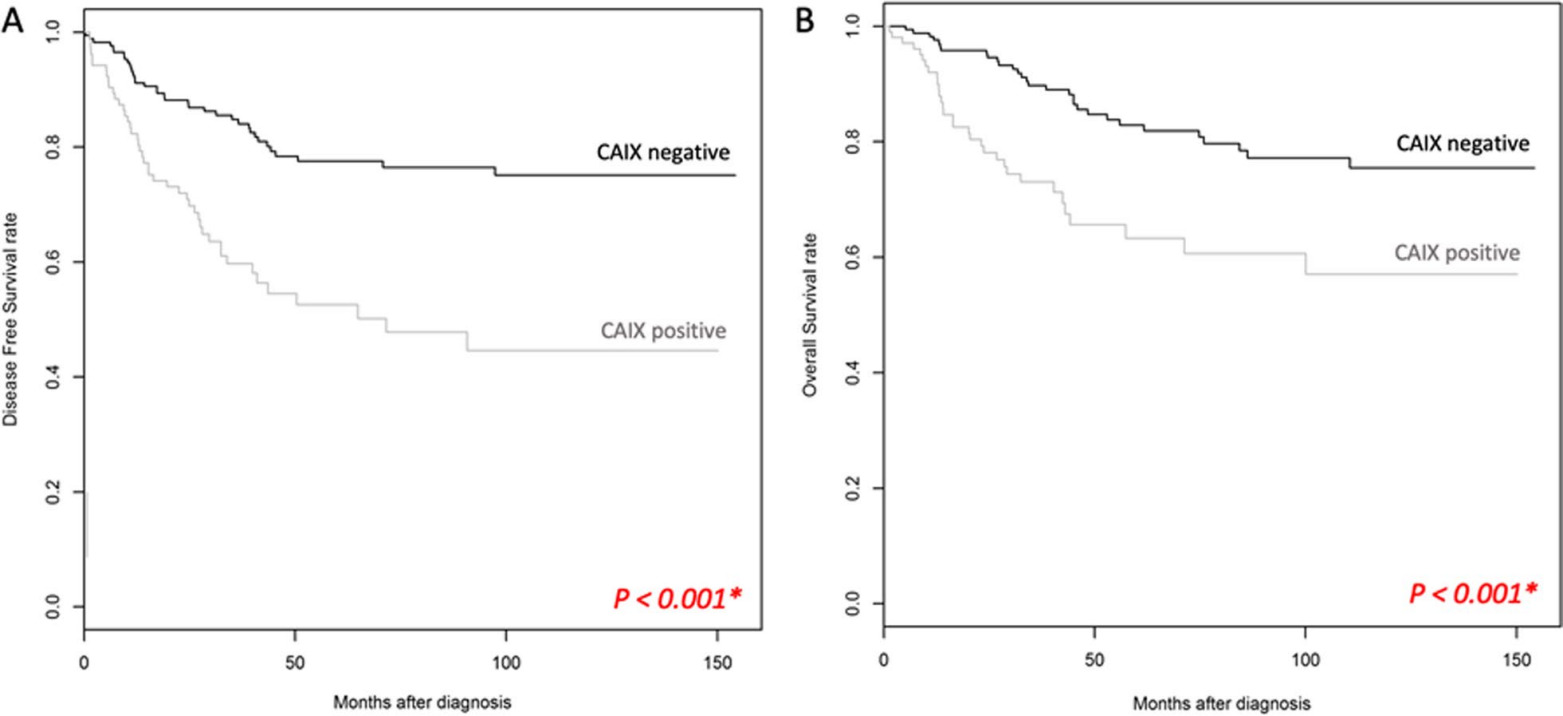
Kaplan–Meier analysis of **A** DFS and **B** OS outcomes in patients with positive CAIX expression

Furthermore, TNBC patients with CAIX-positive expression had significantly worse disease-free survival (DFS) and poorer overall-survival (OS) ([DFS: HR = 2.77, 95% CI 1.78 to 4.31, *P* < 0.001], and [OS: HR = 2.48, 95% CI = 1.50–4.09, *P* < 0.001]) (Fig. 2). Moreover, after adjustment by age, grade, tumour size and lymph node positivity, there is a significant difference between positive CAIX expression and negative CAIX expression in TNBC patients on their survival outcomes ([OS: HR = 2.99, 95% CI = 1.78–5.02, *P* < 0.001], and [DFS: HR = 2.56, 95% CI = 1.41–4.65, *P* = 0.002]).

However, survival analysis for HIF-1α expression in TNBC patients found no statistical differences in DFS and OS ([DFS: *P* = 0.137], and [OS: *P* = 0.807]). Although significant correlation between CAIX and HIF-1α protein expression in tumours was observed (*P* = 0.013) (Table 2); further adjustments by age, grade, tumour size, lymph node stage and HIF-1α H score in survival outcomes of CAIX tumour expression, HIF-1α did not affect CAIX risks on poorer survival and prognostic outcomes ([DFS: HR = 2.95, 95% CI 1.75 to 5.00, *P* < 0.001]*,* and [OS: HR = 2.43, 95% CI 1.34 to 4.41, *P* = 0.004]).

### Co-expression of HIF-1α and CAIX protein in TNBC patients is linked with poorer survival rates

In addition, patients with both HIF-1α and CAIX protein co-expression were more likely to have shorter DFS (HR = 3.07, 95% CI 1.72 to 5.49, *P* < 0.001) and poorer OS (HR = 2.30, 95% CI 1.20 to 4.39, *P* = 0.012) (Table 3). After accounting for age, grade, tumour size and lymph node stage, there is a statistically significant association in patients with both HIF-1α and CAIX protein co-expression and survival outcomes ([DFS: HR = 4.46, 95% CI 2.26 to 8.81, *P* < 0.001], and [OS: HR = 3.30, 95% CI 1.57 to 6.94, *P* = 0.002]) (Table 3).

**Table 3:** Correlation of combinatorial CAIX/HIF-1α tumour cell expression with survival outcomes in patients with TNBC

	Unadjusted				Adjusted^a^			
	No of events	No of patients	HR (95% CI)	*P* value	No of events	No of patients	HR (95% CI)	*P* value
**Disease-free survival (DFS)**								
HIF-1α and CAIX								
HIF-1α negative & CAIX negative	20	98	Reference		15	74	Reference	
HIF-1α negative & CAIX positive	18	46	2.48 (1.31,4.69)	0.0053*	17	39	2.66 (1.3, 5.45)	0.0076*
HIF-1α positive & CAIX negative	15	66	1.08 (0.55,2.1)	0.8273	14	49	1.48 (0.7, 3.1)	0.3012
HIF-1α positive & CAIX positive	27	58	3.07 (1.72,5.49)	0.0002*	24	45	4.46 (2.26, 8.81)	< 0.0001*
**Overall-survival (OS)**								
HIF-1α and CAIX								
HIF-1α negative & CAIX negative	19	98	Reference		15	74	Reference	
HIF-1α negative & CAIX positive	14	46	1.99 (1, 3.97)	0.0513	13	39	1.74 (0.8, 3.8)	0.1636
HIF-1α positive & CAIX negative	11	66	0.8 (0.38, 1.69)	0.5657	10	49	0.94 (0.4, 2.19)	0.8844
HIF-1α positive & CAIX positive	18	57	2.3 (1.2, 4.39)	0.0119*	16	44	3.3 (1.57, 6.94)	0.0016*

^a^Multivariate analysis was adjusted for age, grade, tumour size and lymph node stage

^*^Statistically significant values (*P* < 0.05)

### Expression level of hypoxia CAIX-linked genes (*CAIX, DDIT4, TUBA4*α*, HK2 *and* ARL1, WAS, SETX*) is significantly higher in CAIX-positive and CAIX-negative TNBCs, respectively

Out of the 306 viable CAIX TNBC tumours identified for immunoscoring, 105 “positive” and 152 “negative” tumour samples had NanoString RNA data. Samples from four benign breast tumours were also included in this analysis. Student t-tests with Welch’s correction revealed seven genes (*CAIX,* Carbonic Anhydrase IX; *HK2,* Hexokinase 2; *TUBA4*α*,* Tubulin Alpha 4α; *DDIT4,* DNA damage inducible transcript 4; *SETX,* Senataxin; *WAS,* WASP Actin Nucleation Promoting Factor; *ARL1,* ADP Ribosylation Factor Like GTPase 1) that showed differential expression (*P* < 0.05).

**Fig. 3:**
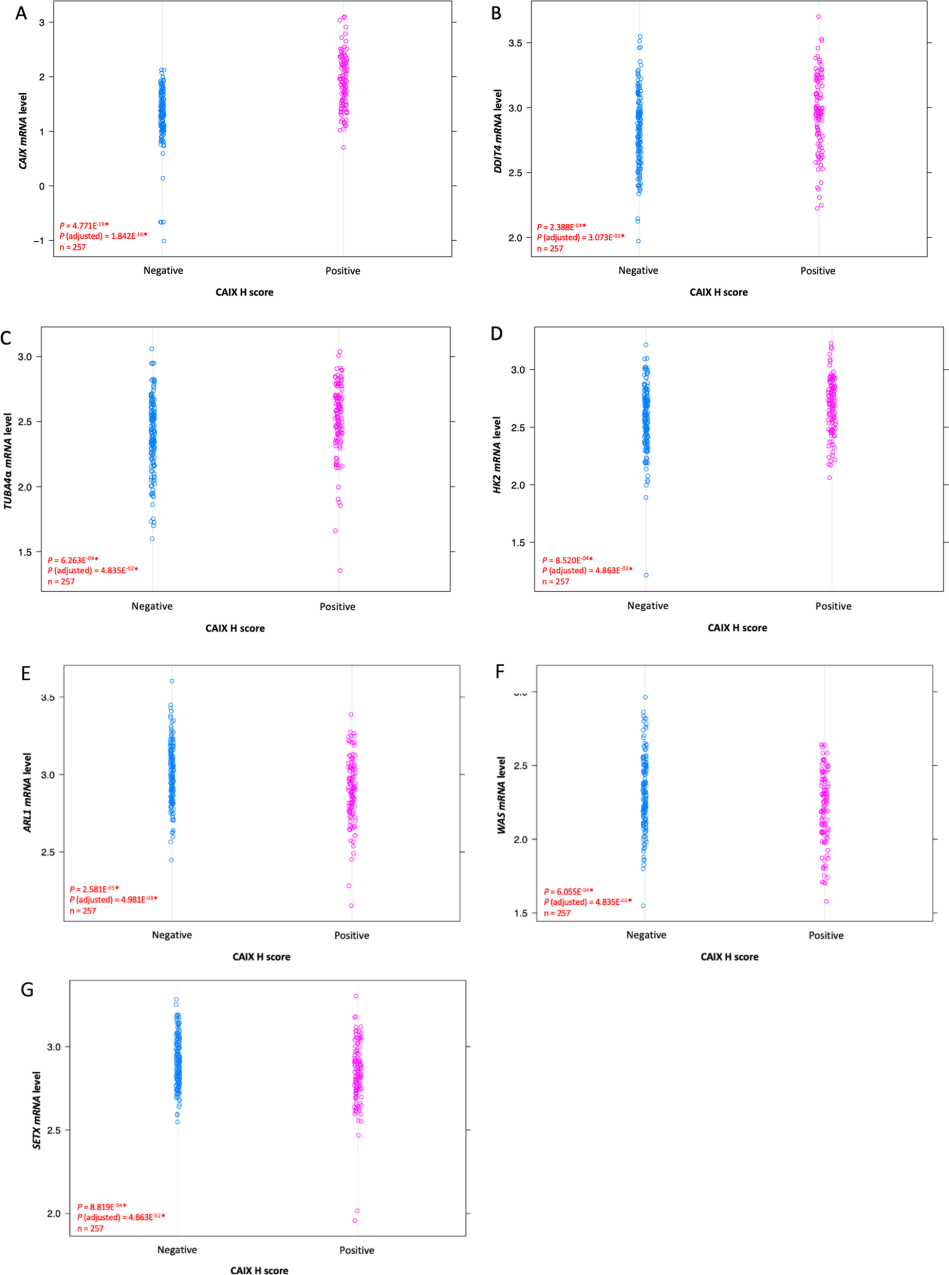
CAIX Strip-plot analysis to mRNA expression of **A** *CAIX*, **B** *DDIT4*, **C** *TUBA4*α, **D** *HK2*, **E** *ARL1*, **F** *WAS*, and **G** *SETX*

**Fig. 4:**
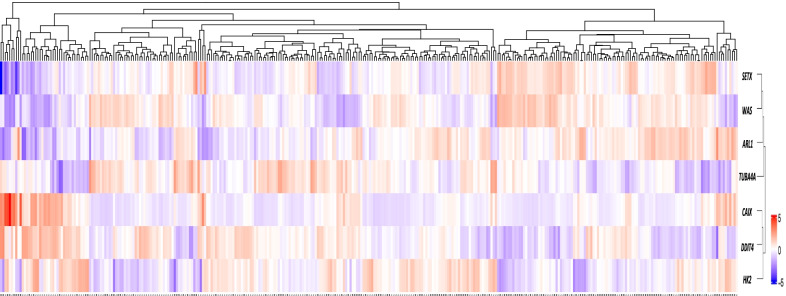
Expression level of a panel of seven significantly DEGs in triple-negative breast cancer (TNBC) patients

Amongst the differentially expressed genes (DEGs), four genes (*CAIX, DDIT4, TUBA4*α*, HK2*) reported significant upregulated expression level in our CAIX-positive TNBC cohort (Fig. 3A–D and Additional file 1: Table S3). On the contrary, the remaining three DEGs (*ARL1, WAS, SETX*) reported significant downregulated expression level in our CAIX-positive TNBC cohort (Fig. 3E–G and Additional file 1: Table S3). Within the seven genes, *CAIX* have been reported to have a similar gene expression profile with *DDIT4* and *HK2* in our TNBC cohort in the heat map (Fig. 4).

### Six differentially expressed genes (*CAIX, HK2, TUBA4*α*, DDIT4, SETX, WAS*) are associated with key cellular pathways modulating tumorigenesis

Gene ontology enrichment analysis identified significant functional enrichment in expression of genes related to secretion (*CAIX, HK2, and TUBA4*α), cellular component disassembly (*DDIT4, HK2, *and* SETX*), regulation of protein complex assembly (*SETX *and* WAS*), glycolytic process (*DDIT4 *and* HK2*), cellular macromolecular complex assembly (*SETX *and* WAS*) and positive regulation of cellular component biogenesis (*SETX *and* WAS*) between the positive and negative CAIX groups in TNBCs. Taken together, these six pathways share six genes which are *CAIX, HK2, TUBA4*α*, DDIT4, SETX *and* WAS* (Table 4).

**Table 4:** Gene ontology enrichment analysis of the seven-gene panel revealed six significantly associated enriched cellular functions

Cellular function and genes	P value
Secretion: *CAIX, HK2, *and* TUBA4*α	0.002*
Cellular component disassembly: *DDIT4, HK2, *and* SETX*	0.004*
Regulation of protein complex assembly: *SETX, *and* WAS*	0.020*
Glycolytic process: *DDIT4, HK2*	0.032*
Cellular macromolecular complex assembly: *SETX, WAS*	0.032*
Positive regulation of cellular component biogenesis: *SETX, WAS*	0.041*

^*^Statistically significant values (*P* < 0.05)

### Low (*WAS, SETX)* and high (*ARL1, DDIT4, TUBA4*α*, CAIX, HK2) mRNA* expression is associated with poorer overall-survival rates in TNBC

Comparison of the prognosis of seven DEGs in TNBC observed that low (*SETX* and *WAS*) and high (*ARL1, DDIT4, TUBA4*α*, CAIX, HK2*) mRNA expression is associated with poorer overall-survival in our SGH TNBC database (*SETX, P* < 0.05; *WAS, P* < 0.001; *ARL1*, *P* = 0.07934; *DDIT4*, *P* < 0.01; *TUBA4*α, *P* = 0.1503; *CAIX*, *P* = 0.2001; *HK2*, *P* = 0.2224) (Table 5, and Additional file 1: Figure S2; Table S4).

**Table 5:** Summary of the comparison between SGH, METABRIC and TCGA patient database for OS

Database	Hypoxia-linked DEGs	DEGs Expression level	Overall survival	P value
SGH	*ARL1*	High	Poor	0.079
METABRIC		High	Poor	0.097
TCGA		High	Poor	0.026*
SGH	*CAIX*	High	Poor	0.200
METABRIC		High	Poor	0.001*
TCGA		High	*Better*	0.091
SGH	*DDIT4*	High	Poor	0.008*
METABRIC		High	Poor	0.073
TCGA		High	*Better*	0.083
SGH	*HK2*	High	Poor	0.222
METABRIC		High	Poor	0.211
TCGA		High	*Better*	0.006*
SGH	*SETX*	Low	Poor	0.035*
METABRIC		Low	Poor	0.140
TCGA		Low	*Better*	0.214
SGH	*TUBA4*α	High	Poor	0.150
METABRIC		High	Poor	0.402
TCGA		High	*Better*	0.016*
SGH	*WAS*	Low	Poor	< 0.001*
METABRIC		Low	Poor	0.010*
TCGA		Low	Poor	0.371

DEGs, Differentially expressed genes

^*^Statistically significant values (*P* < 0.05).

### Comparative survival analysis on DEG expression between SGH, METABRIC and TCGA patient database

Low *WAS* gene expression had poorer OS in all three databases (SGH, *P* < 0.001; METABRIC, *P* < 0.05; TCGA, *P* = 0.3709) (Table 5, and Additional file 1: Figure S2a; Table S4); however, high *WAS* gene expression reported poorer OS post-290 months in the METABRIC database. Moreover, high *ARL1* gene expression also demonstrated poorer overall-survival (OS) in all three databases (SGH, *P* = 0.07934; METABRIC, *P* = 0.09737; TCGA, *P* < 0.05) (Table 5, and Additional file 1: Figure S2b, Table S4). Similarly, high *DDIT4*, high *TUBA4*α*,* high *CAIX,* and high *HK2* gene expression showed poorer OS in SGH and METABRIC databases, respectively (SGH, *P* < 0.01, *P* = 0.1503*, P* = 0.2001, and *P* = 0.2224; METABRIC, *P* = 0.07328, *P* = 0.4021, *P* < 0.001, and *P* = 0.2111) (Table 5, and Additional file 1: Figure S2c, e–g; Table S4).

However, low *DDIT4*, *TUBA4*α*, CAIX,* and *HK2* gene expression reported poorer OS in TCGA database, respectively (*P* = 0.08319, *P* < *0.05*, *P* = 0.09129, and *P* < 0.01) (Table 5, and Additional file 1: Figure S2c, e–g; Table S4).

Furthermore, low *SETX* gene expression had poorer overall-survival (OS) in SGH and METABRIC databases (SGH, *P* < 0.05; METABRIC, *P* = 0.1404) (Table 5, and Additional file 1: Figure S2d; Table S4); however, high *SETX* gene expression reported poorer OS post-290 months in METABRIC database. High *SETX* gene expression had poorer OS in TCGA database (TCGA, *P* = 0.2142) (Table 5, and Additional file 1: Figure S2d; Table S4).

## Discussion

In the present study, we investigated the role of two important hypoxia-regulated markers (HIF-1α and CAIX) and found that increased expression in both CAIX protein and mRNA transcriptional levels are indicators of poorer survival in TNBC. However, HIF-1α protein expression failed to demonstrate any such association with either survival or clinicopathological factors. Interestingly, our results showed that HIF-1α protein expression is not a confounding factor in prognosis of patients expressing CAIX protein. However, co-expression of CAIX and HIF-1α protein in TNBC patients had the poorest prognosis. Furthermore, our study also identified seven CAIX-linked hypoxia genes with prognostic value in our TNBC cohort: *DDIT4, ARL1, WAS, SETX, HK2, TUBA4*α and *CAIX* which have been known to be hypoxia-regulated in vitro.

Our results were in agreement with CAIX protein in breast cancer studies, where 50% of basal-like breast cancers usually have high grade tumours expressing CAIX [22, 23]. Previous clinical studies in invasive breast cancer have also demonstrated the association of CAIX with poor outcome, suggesting that CAIX expression is linked to an aggressive phenotype [11, 16, 24, 25]. Over-expression of CAIX and carbonic anhydrase XII (CAXII) has also been associated with poor DFS in invasive breast cancer. However, the role of CAXII remains unclear and there have been conflicting reports about its role in TNBC. Chen et al. have shown that CAIX correlated with CAXII (*R* = 0.376, *P* = 0.0001) in a cohort of invasive breast cancer [26]. However, our study did not include CAXII and thus, unable show any correlation findings.

Furthermore, our study did not manage to find any prognostic value in HIF-1α protein expression, suggesting that HIF-1α may not be a reliable marker for hypoxia in TNBC. Although there are many markers to assess hypoxia in tumours, such as HIF-1α, X-Box Binding Protein 1 (XBP1), GLUT1 and Vascular endothelial growth factor (VEGF) [7, 8], the results however have been conflicting in various studies. Drawbacks associated with the modification of these hypoxia-responsive protein markers are their potential regulation by non-hypoxia-related factors such as stress, growth factor application, oncogene activation, cell culture densities, local pH, and metabolite concentrations [27]. Therefore, generating hypoxia signatures from in vivo tissue, despite the presence of contaminating stromal tissue, seem to be more robust than those generated from in vitro experiments [28]. Yehia et al. assessed the relative expression of HIF-1α among three breast cancer groups (TNBC, HER2+, ER+/PR+), with TNBC expression results differed only slightly and with little to no statistical significance from the other subgroups, and that HER2 positive tumours showed the highest levels of expression for all studied parameters [29]. This further supports that HIF-1α may not be an exclusive candidate marker for TNBC. Previous findings have demonstrated that HIF-1α was undetectable within minutes after re-oxygenation [30], suggesting that CAIX possibly activates hypoxic condition independently of HIF-1α, as CAIX protein persists longer than HIF-1α. Thus, CAIX as a biomarker for hypoxia could be more suitable as it is more stable and persists longer than HIF-1α.

Moreover, previous findings show that CAIX in high density cultures is induced via the phosphatidylinositol-3-kinase (PI3K) pathway [31] and by the mitogen-activated protein kinase (MAPK) pathway during both normoxia and hypoxia conditions [32]. Taken together, these observations suggest that CAIX expression may also be driven by other HIF-1α-independent signalling pathways to induce hypoxic conditions in the cells. Therefore, CAIX may be a better biomarker for cancer hypoxia.

The seven CAIX-linked hypoxia genes identified in our study have been linked to modulate key functions in tumourigenesis such as DNA repair, metastasis, innate immunity and metabolism in Additional file 1: Table S5. Notably, three of the genes (*DDIT4, WAS, SETX*) are linked to DNA repair functions. DNA damage inducible transcript 4 (DDIT4) acts as an independent prognostic factor for TNBC resistant to neoadjuvant chemotherapy [33]. DDIT4 activity supposedly enhances cancer cell resistance to mTOR inhibitors, thereby increasing cancer cells chemoresistance. Our results further support the notion of significant association between high *DDIT4* mRNA level with poor survival, and reported upregulation in *DDIT4* expression in our CAIX-positive TNBC cohort. Induced DDIT4 expression under cellular stressors and other chemical molecules (e.g. glucocorticoids, endoplasmic reticulum stress inducers, etc.) suggests its role in DNA repair under hypoxic conditions [34].

In the other two genes (*WAS*, *SETX*) linked to DNA repair functions, both downregulated *WAS* and *SETX* mRNA expression is associated with poorer overall-survival. Similarly, a subset of TNBC with increased expression of *WAS* and *SETX* mRNA showed better survival in other studies [35, 36]. Gene *SETX* role in tumourigenesis has been linked to its function in maintaining genome integrity via the coordination of transcription, DNA replication and DNA damage response [35], whereas gene *WAS* encodes for the cytoskeletal regulator, Wiskott-Aldrich syndrome protein (WASP), which plays a key role in tumourigenesis via binding to double strand breaks, regulating RNA Polymerase II activity and facilitating actin polymerization [37]. Its influence on actin filament dynamics and facilitation of actin reorganization, such as branching and crosslinking, are inherent in metastasis and invasion [37, 38]. Moreover, WASP and Arp2/3 complex have been reported to be recruited to damaged DNA double-strand breaks sites to promote double-strand breaks clustering and homology-directed repair [38, 39].

Thus, these further supports that the integrity of DNA-repair mechanism may be essential for protection against hypoxia-mediated DNA damage [36, 40, 41]. These biological categories have known functional relationships on breast cancer development and the aforementioned genes’ value as diagnostic markers and therapeutic targets deserves further investigation.

Within our seven gene DEG signature, *TUBA4*α is linked to metastasis, *HK2* and *CAIX* is linked to promoting tumourigenesis, while the remaining *ARL1* is linked to innate immunity [42]. Our results showed that these four genes were upregulated within the CAIX-positive group and associated with poorer survival outcomes in this subset of TNBC patients. Upregulation of *TUBA4*α disrupts the optimal tubulin isotype compositions in cell [43] and the dynamics of microtubule polymerisation and depolymerisation are of key importance in spindle formation during mitosis [44]. Moreover, upregulation of *HK2* drives glucose metabolism and promotes sufficient number of metabolic intermediates to support anabolic processes (such as nucleic acid, lipid and protein synthesis), which is characteristic of rapidly dividing cancer cells [45]. While upregulation of *CAIX* disrupts pH balance [46], resulting in a hypoxic environment, which is also regulated under hypoxic condition through the hypoxia inducible factor (HIF1) cascade, promoting tumorigenesis. Thus, these genes are associated with aggressive cancer features and proliferation within the tumour microenvironment, reflecting the poorer survival outcome in our study.

Our study has several limitations. Since the FFPE blocks used in TMA construction were dated from 2003 to 2013, the tissue quality may be considered a limitation of this study. Tissue quality may contribute to the reduction of antigenicity and decrease in the sensitivity of the IHC reaction, leading to reduced protein detection. Furthermore, the FFPE tissue quality may also affect the amount of viable RNA for NanoString extraction and experiments. Although this study was conducted on a limited number of patient samples, the data indicates that quantification of hypoxia-related genes in TNBC can have potential prognostic value regardless of treatment type. Moreover, it is imperative that the clinical relevance of the seven hypoxia-linked gene signatures to be validated in independent studies with larger patient cohorts. Protein expression of the aforementioned genes showing significant association with survival is being studied in ongoing follow-up studies.

## Conclusion

In conclusion, our study demonstrated that CAIX expression is independently associated with a poorer clinical and survival outcome in TNBC. Since hypoxia is increasingly being studied for being responsible for resistance against radiotherapy and emerging immunotherapy [47], the identification of the seven-genes associated with CAIX could be a step forward to test for hypoxia in TNBCs and possibly improve patients’ treatment regimen and prognosis. Thus, further studies on the seven-gene hypoxia panel are warranted.

## Supplementary Information

**Additional file 1.** Document contains supplementary tables and figures mentioned in the manuscript

## Data Availability

The data that support the findings of this study are available from the Molecular Taxonomy of Breast Cancer International Consortium (METABRIC) and The Cancer Genome Atlas (TCGA) were obtained from cBioPortal (http://www.cbioportal.org/). The datasets generated and analysed during the current study are not publicly available due restrictions from institutional policy on human tissue data but are available from the corresponding author on reasonable request.

## Abbreviations

TNBC: Triple-negative breast cancer
ER: Oestrogen receptor
PR: Progesterone receptor
HER2: Human epidermal growth factor receptor 2
HIF-1: Hypoxia inducible factor 1
CAIX: Carbonic anhydrase IX
GLUT1: Glucose transporter 1
FFPE: Formalin-fixed paraffin-embedded
TMA: Tissue Microarray
IHC: Immunohistochemical staining
DAB: Diaminobenzidine substrate
GO: Gene ontology
DFS: Disease-free survival
OS: Overall survival
DEG: Differentially expressed genes
HK2: Hexokinase 2
TUBA4α: Tubulin Alpha 4α
DDIT4: DNA damage inducible transcript 4
SETX: Senataxin
WAS: WASP Actin Nucleation Promoting Factor
ARL1: ADP Ribosylation Factor Like GTPase 1
CAXII: Carbonic anhydrase XII
XBP1: X-Box Binding Protein 1
VEGF: Vascular endothelial growth factor
PI3K: Phosphatidylinositol-3-kinase
MAPK: Mitogen-activated protein kinase
WASP: Wiskott-Aldrich syndrome protein
