# Supplementary material for: Hypoxia-regulated carbonic anhydrase IX (CAIX) protein is an independent prognostic indicator in triple negative breast cancer
Source: Breast Cancer Res. 2022 Jun 3;24:38. doi: 10.1186/s13058-022-01532-0 (PMC9164406; doi:10.1186/s13058-022-01532-0)
Supplement: Supplementary file 1 — Additional file 1. Document contains supplementary tables and figures mentioned in the manuscript [file 13058_2022_1532_MOESM1_ESM.docx]

**Additional File 1 – Supplementary Materials**

***Supplementary* Table 1.** Details of antibodies used for immunohistochemistry (IHC) of triple-negative breast cancer sections

| **Antibody** | **Clone** | **Dilution** | **Source** | **Cellular localization** | **Antigen retrieval** |
| --- | --- | --- | --- | --- | --- |
| HIF-1α (IHC) | EP1215Y/IgG | 1:200 | Abcam ab51608 | Tumour cells, cytoplasm (normoxia); nucleus (hypoxia) | ER1 (20 minutes) |
| CAIX (IHC) | Ep161/IgG | 1:100 | Cell Marque 379R-16 | Tumour cells, membranous | SC1 |

IHC, Immunohistochemistry; CAIX, Carbonic Anhydrase IX; HIF-1α, Hypoxia Inducible Factor 1 Subunit Alpha; Cell Marque (Cell Marque, Rocklin, CA, USA); Abcam (Abcam, Cambridge, MA, USA)

**Supplementary Table 2.** Comparison of clinicopathological features and characteristics of TNBC patients bearing positive or negative HIF-1α tumour cell expression

|  | **HIF-1α** | | |
| --- | --- | --- | --- |
| **Factors** | HIF-1α Negative | HIF-1α Positive | P value |
| **Age at Diagnosis^ (years)** | 54 (45.5, 62) | 57 (50, 63) | 0.061 |
| **Ethnicity** |  |  | 1.000 |
| Chinese | 130 (81.8%) | 115 (81.6%) |  |
| Indian | 9 (5.7%) | 8 (5.7%) |  |
| Malay | 8 (5.0%) | 7 (5.0%) |  |
| Others | 12 (7.5%) | 11 (7.8%) |  |
| **Laterality** |  |  | 0.820 |
| Left | 86 (51.8%) | 72 (50.3%) |  |
| Right | 80 (48.2%) | 71 (49.7%) |  |
| **Histological Grade** |  |  | 0.754 |
| 1/2 | 27 (16.5%) | 21 (14.8%) |  |
| 3 | 137 (83.5%) | 121 (85.2%) |  |
| **Tumour Size 20mm** |  |  | 0.908 |
| ≤20mm | 50 (31.1%) | 42 (30.4%) |  |
| >20mm | 111 (68.9%) | 96 (69.6%) |  |
| **Lymphovascular Invasion** |  |  | 0.267 |
| No | 94 (61.4%) | 92 (68.1%) |  |
| Yes | 59 (38.6%) | 43 (31.9%) |  |
| **Lymph node positivity** |  |  | 0.681 |
| Absent | 70 (58.3%) | 54 (55.1%) |  |
| Present | 50 (41.7%) | 44 (44.9%) |  |
| **Tumour Borders** |  |  | 1.000 |
| Infiltrative | 112 (94.9%) | 92 (94.8%) |  |
| Pushing | 6 (5.1%) | 5 (5.2%) |  |

^Age is presented as median (Interquartile range)

***Supplementary*** **Table 3.** Supplementary statistical data on the strip-plot analysis comparing between CAIX-positive and CAIX-negative H score groups versus mRNA expression level of seven differentially expressed hypoxic CAIX-linked genes.

| **Hypoxia-linked DEGs** | **H score** | **N** | **Mean expression difference between CAIX positive/negative H score groups** | **Lower 95% CI** | **Upper 95% CI** | **Test statistics** | **Nominal P value** | **Adjusted P value** |
| --- | --- | --- | --- | --- | --- | --- | --- | --- |
| *CAIX* | CAIX | 257 | -0.61756472 | -0.74219364 | -0.49293581 | -9.76371563 | **4.7708E^-19^*** | **1.8415E^-16^*** |
| *ARL1* | CAIX | 257 | 0.11181782 | 0.06059041 | 0.16304523 | 4.30288853 | **2.5811E^-05^*** | **0.0049815*** |
| *DDIT4* | CAIX | 257 | -0.13725889 | -0.20967697 | -0.06484081 | -3.73540833 | **0.00023882*** | **0.03072876*** |
| *WAS* | CAIX | 257 | 0.10863268 | 0.04709287 | 0.17017249 | 3.47861916 | **0.00060554*** | **0.04834684*** |
| *TUBA4*α | CAIX | 257 | -0.12158082 | -0.19063011 | -0.05253153 | -3.47027562 | **0.00062625*** | **0.04834684*** |
| *HK2* | CAIX | 257 | -0.10895818 | -0.17249496 | -0.04542139 | -3.37828551 | **0.00085201*** | **0.04863093*** |
| *SETX* | CAIX | 257 | 0.0774549 | 0.03226338 | 0.12264642 | 3.38176336 | **0.00088191*** | **0.04863093*** |

* Statistically significant values (p<0.05). DEGs, Differentially expressed genes; CI, Confidence Interval.

***Supplementary*** **Table 4.** Supplementary statistical data on the comparison between SGH, METABRIC and TCGA patient database for overall survival (OS)

| **Database** | **Hypoxia-linked DEGs** | **Threshold percentile** | **Threshold** | **Z-score** | **P value** | **Lower**  **95% CI** | **Upper**  **95% CI** | **Log-rank test**  **p-value** | **Log-rank**  **test** | **N** |
| --- | --- | --- | --- | --- | --- | --- | --- | --- | --- | --- |
| SGH | *ARL1* | 0.8 | 3.10934762 | 1.75452353 | 0.07934084 | 0.94870856 | 2.59091057 | 0.0880253 | 2.9101204 | 317 |
| METABRIC |  | 0.6 | 8.8011555 | 1.65775623 | 0.09736668 | 0.95366781 | 1.76455992 | 0.09928816 | 2.71694059 | 320 |
| TCGA |  | 0.7 | 0.63595714 | 2.21886462 | **0.02649594*** | 1.130063 | 7.19634878 | 0.0298932 | 4.71542352 | 98 |
| SGH | *CAIX* | 0.8 | 1.97783565 | 1.28129548 | 0.2000899 | 0.82405634 | 2.51989379 | 0.21557299 | 1.53359975 | 317 |
| METABRIC |  | 0.6 | 6.49838017 | 3.38764237 | **0.00070496*** | 1.24803942 | 2.29318495 | 0.0008126 | 11.2122206 | 320 |
| TCGA |  | 0.5 | -2.17255556 | -1.68862097 | 0.09129209 | 0.14599709 | 1.1538443 | 0.07521258 | 3.16543112 | 98 |
| SGH | *DDIT4* | 0.8 | 3.1277963 | 2.66570866 | **0.00768262*** | 1.2108879 | 3.50501079 | 0.01194616 | 6.31884154 | 317 |
| METABRIC |  | 0.2 | 9.15340207 | 1.79106472 | 0.07328291 | 0.96601678 | 2.15511566 | 0.06264454 | 3.46594925 | 320 |
| TCGA |  | 0.3 | -0.72386 | -1.73246521 | 0.08319077 | 0.17386421 | 1.11381314 | 0.08730812 | 2.92331895 | 98 |
| SGH | *HK2* | 0.4 | 2.568181 | 1.22010612 | 0.22242465 | 0.82984289 | 2.22932481 | 0.21580778 | 1.5320317 | 317 |
| METABRIC |  | 0.8 | 7.61620368 | 1.25049385 | 0.2111192 | 0.87533598 | 1.82670203 | 0.22125445 | 1.4962164 | 320 |
| TCGA |  | 0.4 | -0.57244286 | -2.74895429 | **0.00597857*** | 0.06674781 | 0.63537726 | 0.00243669 | 9.18753383 | 98 |
| SGH | *SETX* | 0.3 | 2.80163336 | -2.11193639 | **0.03469191*** | 0.37009454 | 0.96358153 | 0.03882944 | 4.26831397 | 317 |
| METABRIC |  | 0.7 | 6.88986096 | -1.47425368 | 0.1404133 | 0.54864051 | 1.08861219 | 0.1330091 | 2.25702808 | 320 |
| TCGA |  | 0.5 | -0.0081 | 1.24203696 | 0.21422293 | 0.70526757 | 4.74625812 | 0.207449 | 1.58914036 | 98 |
| SGH | *TUBA4*α | 0.6 | 2.52890087 | 1.43848149 | 0.15029748 | 0.88249748 | 2.25828871 | 0.15262912 | 2.04577621 | 317 |
| METABRIC |  | 0.5 | 7.47433519 | 0.83786746 | 0.40210515 | 0.84044872 | 1.54249391 | 0.40167518 | 0.70330563 | 320 |
| TCGA |  | 0.5 | 0.01225 | -2.41427639 | **0.0157665*** | 0.08313939 | 0.77234298 | 0.00774014 | 7.09263506 | 98 |
| SGH | *WAS* | 0.3 | 2.1490429 | -4.06849597 | **4.7318E^-05^*** | 0.23611545 | 0.60359014 | 7.028E-05 | 15.8033032 | 317 |
| METABRIC |  | 0.7 | 9.58506717 | -2.56646342 | **0.01027415*** | 0.43934292 | 0.89565136 | 0.00770127 | 7.10165694 | 320 |
| TCGA |  | 0.2 | 0.0828 | -0.89471635 | 0.37093874 | 0.23722469 | 1.71066225 | 0.38405497 | 0.7576818 | 98 |

* Statistically significant values (p<0.05). DEGs, Differentially expressed genes; CI, Confidence Interval

**Supplementary Table 5.** List of differentially expressed hypoxic CAIX-linked genes and their function on tumourigenesis

| **Gene** | **Function on tumourigenesis** |
| --- | --- |
| *ARL1* | **Innate immunity**  (1) |
| *DDIT4* | **DNA repair**  (2) |
| *HK2* | **Metabolism**  (3) |
| *SETX* | **DNA repair**  (4) |
| *WAS* | **DNA repair**  (5-7) |
| *CAIX* | **Metabolism**  (8) |
| *TUBA4*α | **Metastasis**  (9, 10) |

**Supplementary Figure 1.** Volcano plot on the identification of seven significantly differentially-expressed genes (DEGs) among the NanoString gene panel


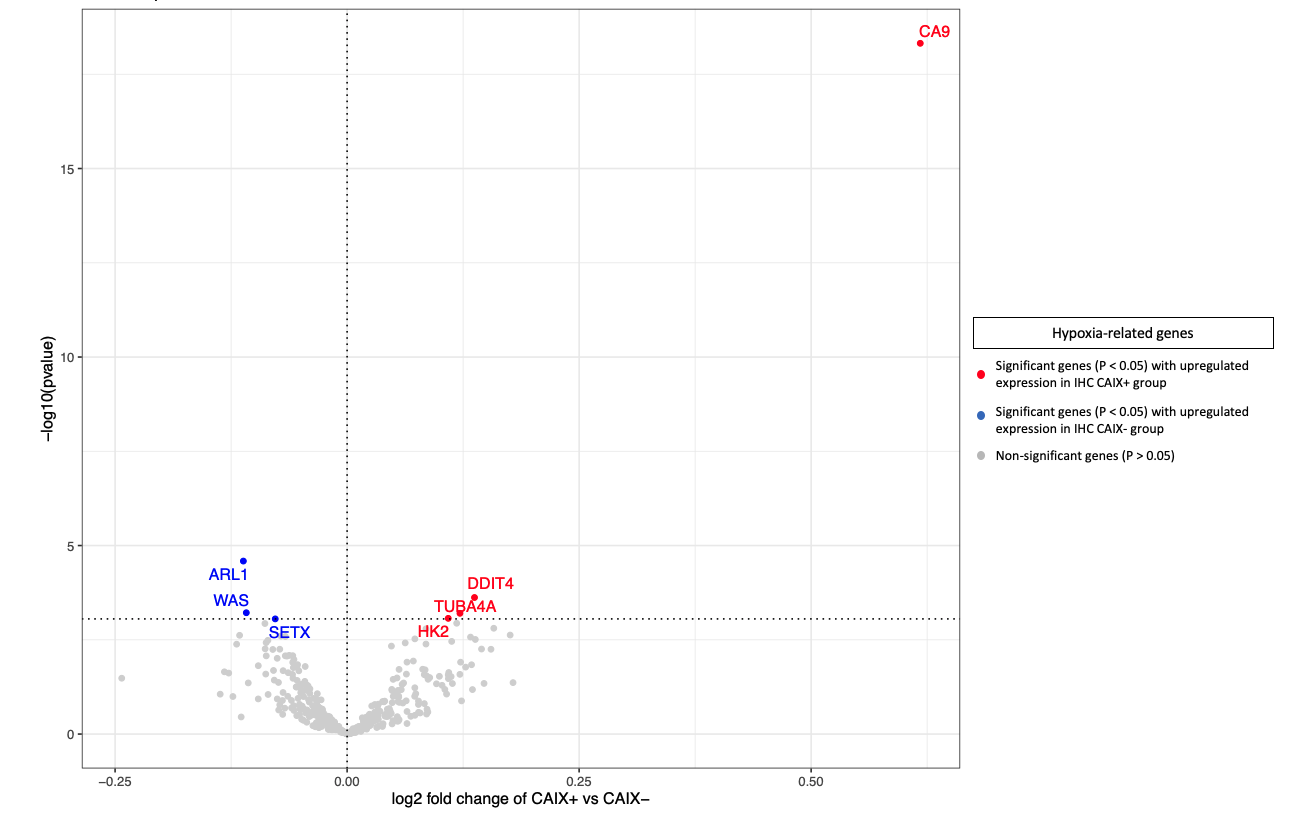


**Supplementary Figure 2.** Comparison of SGH, METABRIC and TCGA patient database on OS for A) *WAS* genes expression, B) *ARL1* genes expression, C) *DDIT4* genes expression, D) *SETX* genes expression, E) *TUBA4*α genes expression, F) *CAIX* genes expression and G) *HK2* genes expression


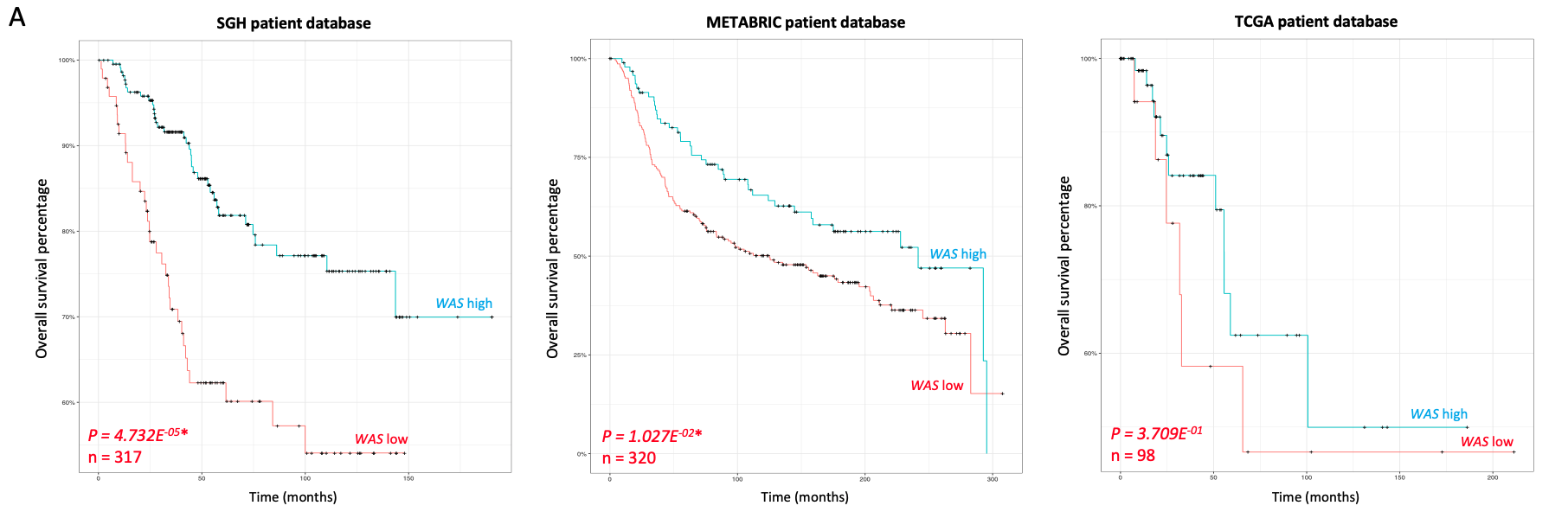


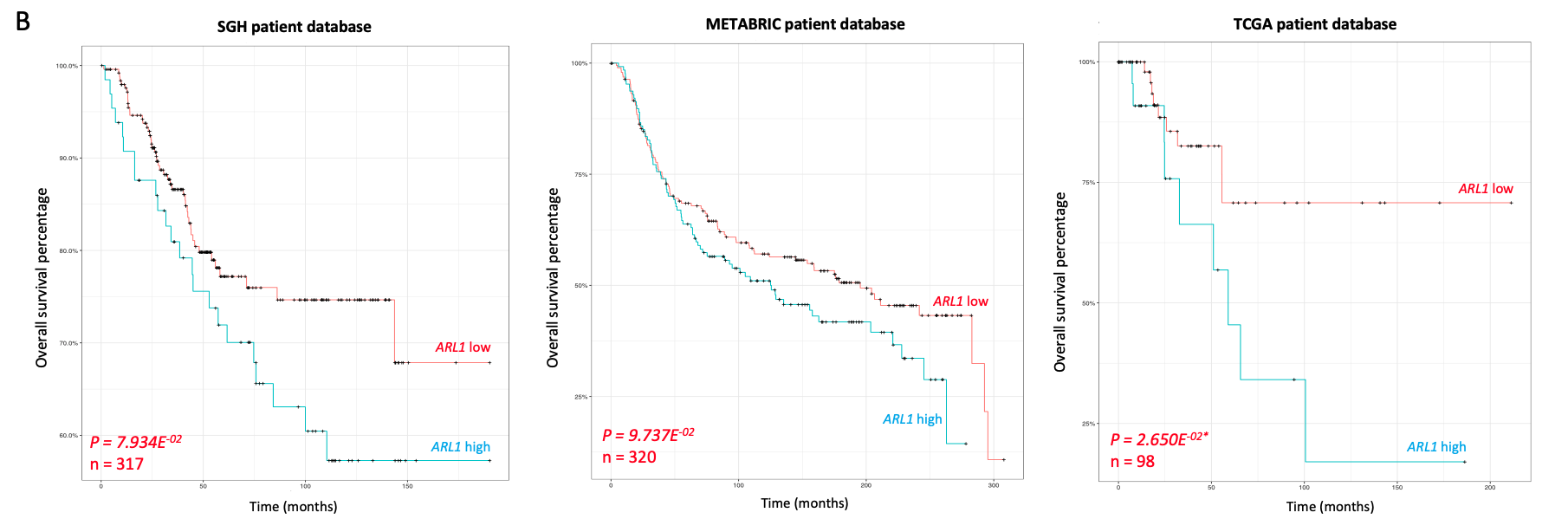


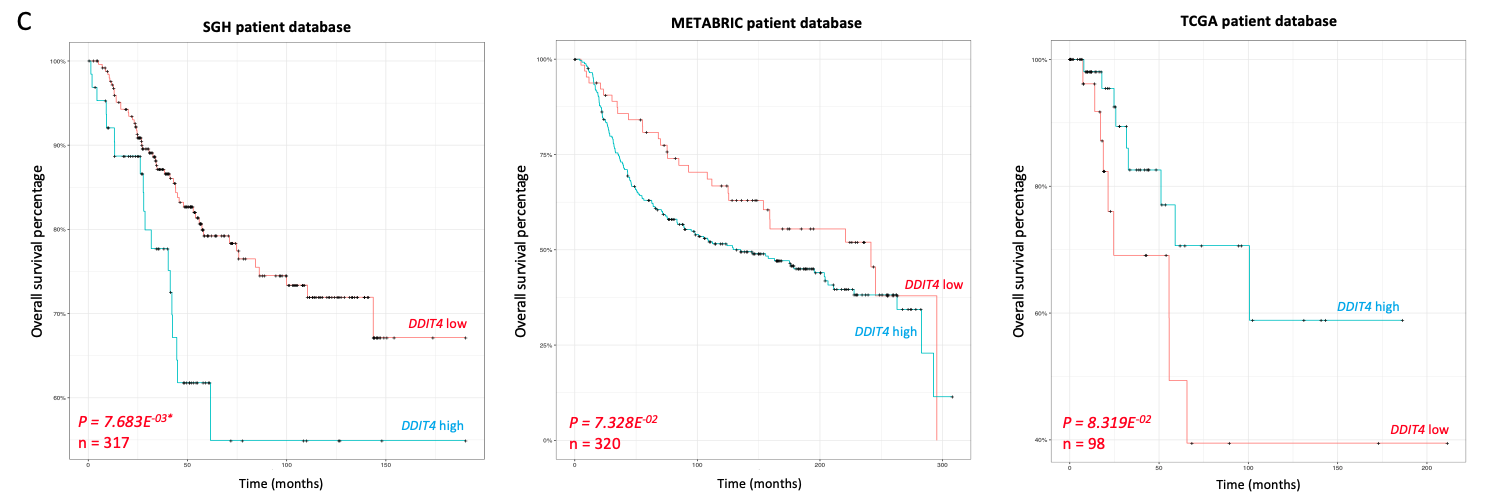


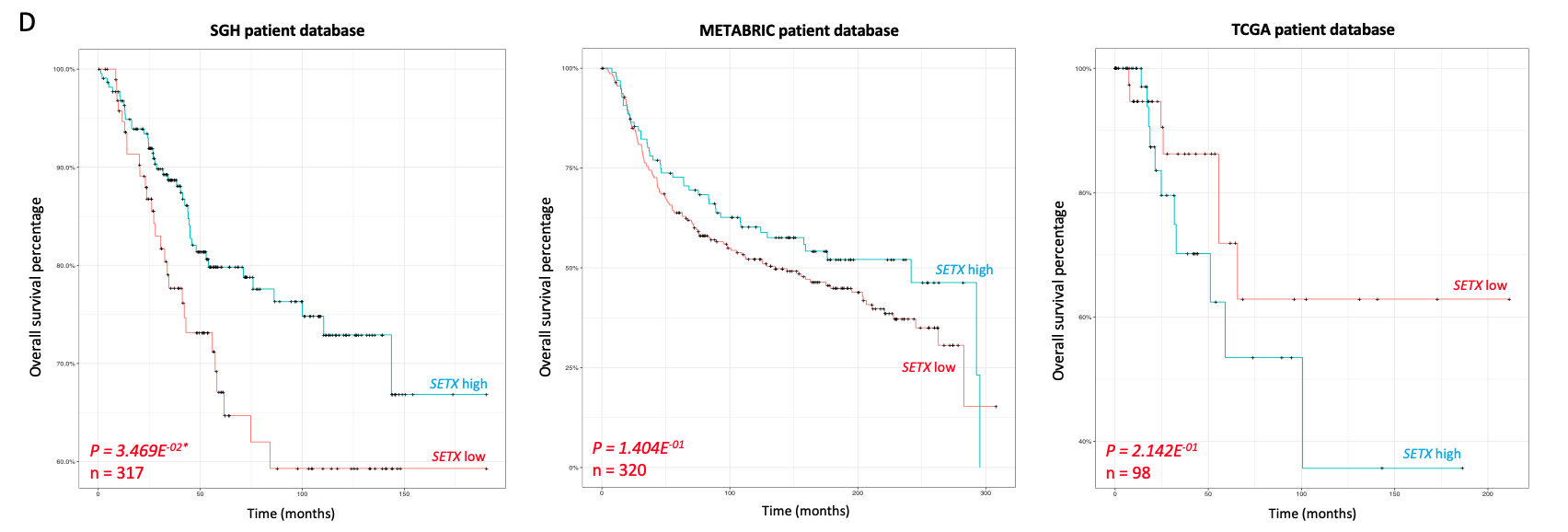


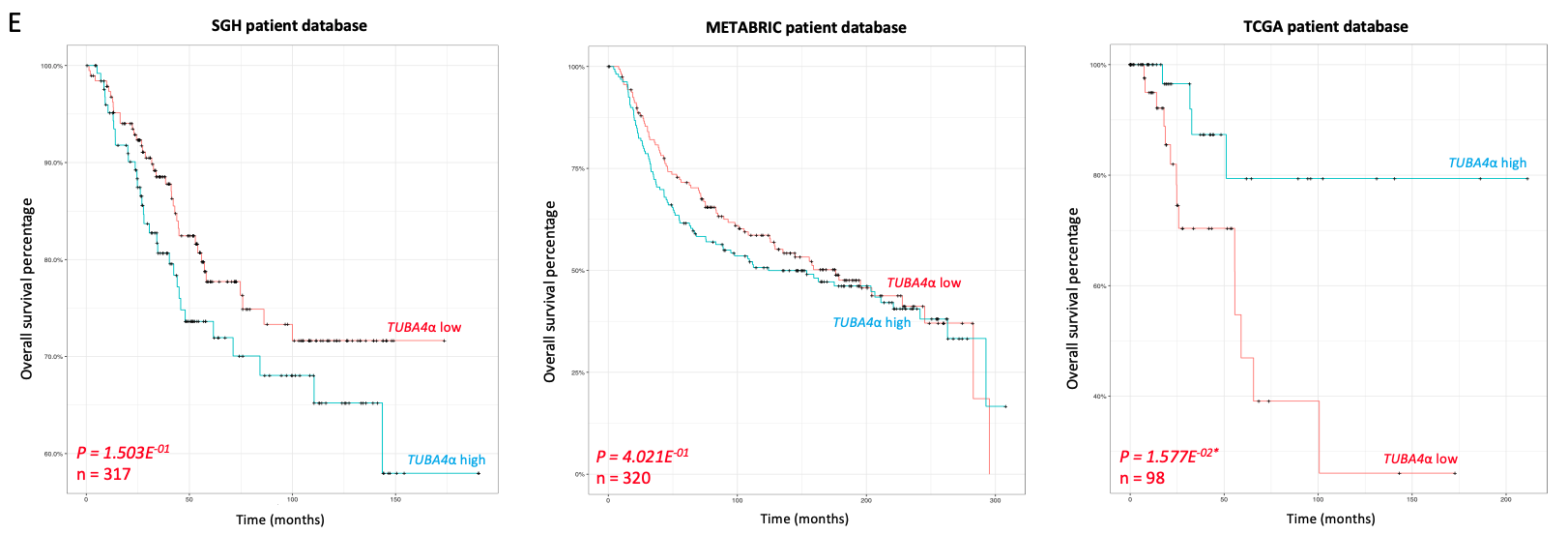


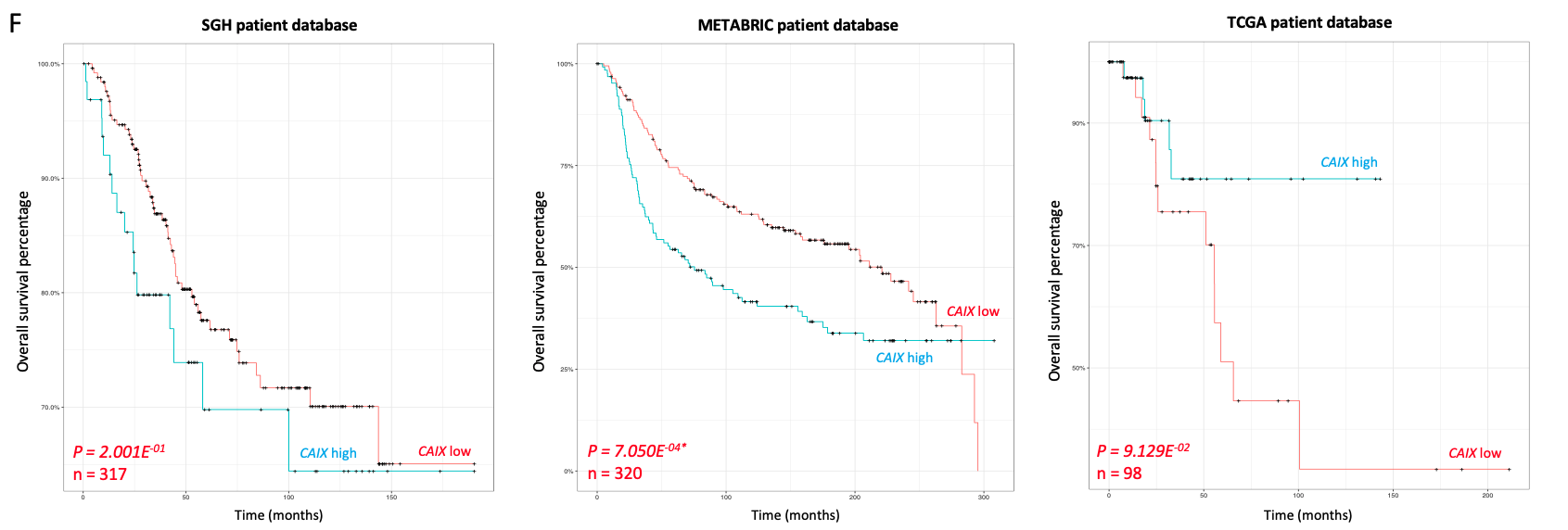


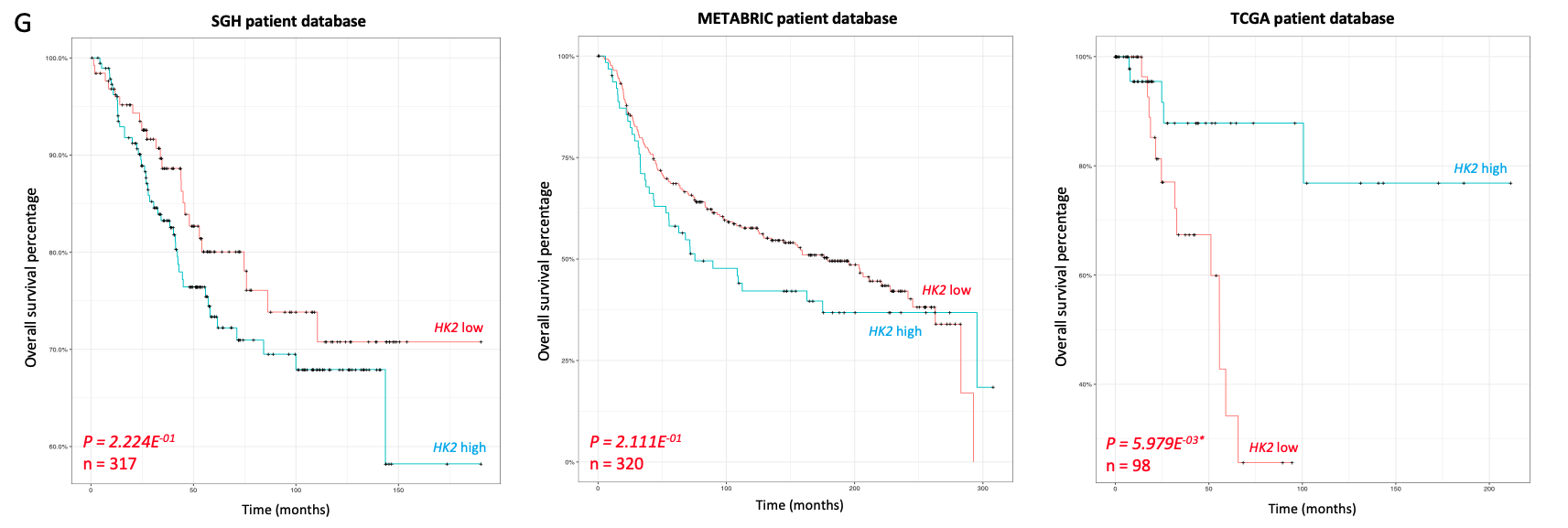


References

1. Lu L, Tai G, Hong W. Autoantigen Golgin-97, an effector of Arl1 GTPase, participates in traffic from the endosome to the trans-golgi network. Mol Biol Cell. 2004;15(10):4426-43.

2. Tirado-Hurtado I, Fajardo W, Pinto JA. DNA Damage Inducible Transcript 4 Gene: The Switch of the Metabolism as Potential Target in Cancer. Front Oncol. 2018;8:106.

3. Patra KC, Wang Q, Bhaskar PT, Miller L, Wang Z, Wheaton W, et al. Hexokinase 2 is required for tumor initiation and maintenance and its systemic deletion is therapeutic in mouse models of cancer. Cancer Cell. 2013;24(2):213-28.

4. Becherel OJ, Yeo AJ, Stellati A, Heng EY, Luff J, Suraweera AM, et al. Senataxin plays an essential role with DNA damage response proteins in meiotic recombination and gene silencing. PLoS Genet. 2013;9(4):e1003435.

5. Hurst V, Shimada K, Gasser SM. Nuclear Actin and Actin-Binding Proteins in DNA Repair. Trends Cell Biol. 2019;29(6):462-76.

6. Izdebska M, Zielińska W, Hałas-Wiśniewska M, Grzanka A. Involvement of Actin and Actin-Binding Proteins in Carcinogenesis. Cells. 2020;9(10).

7. Schrank BR, Aparicio T, Li Y, Chang W, Chait BT, Gundersen GG, et al. Nuclear ARP2/3 drives DNA break clustering for homology-directed repair. Nature. 2018;559(7712):61-6.

8. Supuran CT. Carbonic anhydrases: novel therapeutic applications for inhibitors and activators. Nat Rev Drug Discov. 2008;7(2):168-81.

9. Nami B, Wang Z. Genetics and Expression Profile of the Tubulin Gene Superfamily in Breast Cancer Subtypes and Its Relation to Taxane Resistance. Cancers (Basel). 2018;10(8).

10. Parker AL, Teo WS, McCarroll JA, Kavallaris M. An Emerging Role for Tubulin Isotypes in Modulating Cancer Biology and Chemotherapy Resistance. Int J Mol Sci. 2017;18(7).
